# Structural intervention proposals and the delegitimization of the institutions involved

**DOI:** 10.1093/eurpub/ckac131.530

**Published:** 2022-10-25

**Authors:** T van Meurs, J Oude Groeniger, W de Koster, J van der Waal

**Affiliations:** 1 Department of Public Administration and Sociology, Erasmus University Rotterdam, Rotterdam, Netherlands; 2 Department of Public Health, Erasmus MC, Rotterdam, Netherlands

## Abstract

Improving citizens’ nutritional intake is crucial to combat obesity. Especially structural interventions are effective; likely due to bypassing issues of individual agency. However, such restrictive interventions have low public acceptance, and pushback may create a negative feedback loop, adversely impacting the perceived legitimacy of the institutions involved. This may be especially so among less-educated individuals, as they consider health promotion institutions less legitimate. Recent theorizing argues this may be due to the cultural distance perceived towards those populating the institutions. As structural interventions mainly target behavior more prevalent among less-educated individuals - e.g. sugar-sweetened beverage (SSB) consumption - this perceived distance may be enhanced. With a preregistered survey-experiment among a high-quality representative sample, fielded in February ‘22, we test if proposed structural interventions affect perceived legitimacy of health promotion institutions, and if this differs between educational groups. We used the strategically selected case of interventions to reduce SSB consumption in the Netherlands - currently not tackled by official interventions. This allowed us to confront citizens with a potential - rather than implemented - intervention, singling out its effects more clearly. Respondents were randomized into i) a control group presented with an explanation of the institutions and their information provision approach; or a treatment group with this information, plus ii) a proposed sugar tax or iii) a proposed regulated decrease of sugar content. Results indicate that institutions are perceived as less legitimate when citizens are confronted with structural intervention proposals, but little evidence for a moderating role of education is found. These results can improve awareness among policymakers regarding interventions effects besides health outcomes, and their risk for delegitimizing health institutions.

**Key messages:**

• Proposals for structural interventions decrease the perceived legitimacy of involved health promotion institutions across society.

• Less-educated citizens perceive health promotion institutions as less legitimate at baseline; structural intervention proposals maintain and occasionally increase this gap.

